# Cone-Beam Computed Tomography-Based Investigation of the Prevalence and Distribution of Pulp Stones and Their Relation to Local and Systemic Factors in the Makkah Population: A Cross-Sectional Study

**DOI:** 10.7759/cureus.51633

**Published:** 2024-01-04

**Authors:** Laila M Kenawi, Haytham S Jaha, Mashael M Alzahrani, Jihan I Alharbi, Shahad F Alharbi, Taif A Almuqati, Rehab A Alsubhi, Wahdan M Elkwatehy

**Affiliations:** 1 Department of Restorative Dentistry, Faculty of Dental Medicine, Umm Al-Qura University, Makkah, SAU; 2 Department of Endodontics, Faculty of Dentistry, Cairo University, Cairo, EGY; 3 Department of Dentistry, Umm Al-Qura University, Makkah, SAU; 4 Department of Dental Public Health and Preventive Dentistry, Faculty of Dentistry, Mansoura University, Mansoura, EGY

**Keywords:** dental pulp calcification, saudi arabia, makkah, pulp stone, cone-beam computed tomography

## Abstract

Objectives

This study aimed to assess the prevalence and distribution of dental pulp stones and evaluated their possible associations with local and systemic factors in the Makkah population in Saudi Arabia.

Materials and methods

Archived cone-beam computed tomography (CBCT) images for 390 patients, from the electronic health records (EHR) in the Dental Teaching Hospital, Umm Al-Qura University, were used. Images were examined in all planes (coronal, sagittal, and axial) for a discrete radiopaque mass in the pulp of all teeth, in both arches. The teeth conditions (the presence of caries, restoration, the periodontal condition, and the presence of pulp stones) were recorded. Additional patient information, including age, gender, and medical condition, was obtained from the patients' archived files. The collected data were statistically analyzed using the Statistical Package for Social Sciences (SPSS) (IBM SPSS Statistics, Armonk, NY) software; a p-value of ≤ 0.05 is considered statistically significant.

Results

Pulp stone prevalence was 78.97% of the subjects (308 out of 390) and 15.92% of the examined teeth (1644 out of 10326). There were statistically significant differences regarding nationality (p=0.043) and age (p=0.023) but no significant difference between males and females (p=0.876), maxillary and mandibular teeth (p=0.392), and right and left sides (p=0.222) in pulp stone prevalence. Significant differences were found between pulp stone prevalence of sound versus and carious and restored teeth and between periodontally affected teeth and periodontally healthy teeth (p=0.031).

Conclusion

The prevalence of pulp stones in the Makkah population is high. A positive association was found between nationality, age, tooth restorations, caries, periodontal diseases, and pulp stone prevalence, but no correlation was found with patients' health or gender. The molars were the most affected teeth, while the incisors were the least.

## Introduction

Dental pulps may contain calcified substances known as "pulp stones." They are visible in healthy, damaged, and even unerupted teeth [1]. They are more frequent in the coronal than in the radicular pulp [2]. All teeth can have pulp stones; however, the molars are known to be the most commonly affected [3].

Pulp stones are typically asymptomatic unless invading any nerve fiber bundles [4]. A few etiological factors, including aging; genetic predisposition; long-lasting irritants such as caries, deep fillings, chronic inflammation, and abrasion; orthodontic tooth movement; trauma; periodontal disease; medications; anemia, arteriosclerosis; acromegaly; and Marfan syndrome, have been put forth; however, the exact cause of pulp stone is unknown [5].

According to Edds et al. [6] in 2005, significantly more individuals with pre-existing cardiovascular diseases (CVD) developed identifiable pulp stones. Another study showed that in those who had undergone kidney transplantation, pulp and carotid artery calcifications were not correlated [7]. This was completely in conflict with Näsström et al. [8] who found a positive correlation. Tassoker et al. [5] in 2018 reported that 3.8% of the 105 individuals with pulp stones had a systemic disease, such as a cardiovascular, endocrine, or pulmonary condition.

Researchers have examined pulp stones using a variety of radiographic methods, including bitewing, intraoral periapical radiographs, and orthopantomography (OPG) [9-11]. ‏Pulp stones appear as radiopaque structures in the pulp chamber or root canal with different sizes, shapes, and numbers. Some may fill the pulp chamber; others could have a diameter of 2 or 3 mm [12].

Cone-beam computed tomography (CBCT) helps to detect pulp stones in several aspects without the limitations of traditional radiography and histological evaluation. For the easier identification of pulp stones in radicular pulp, CBCT may be a sensitive tool to identify them [13].

Pulp calcification may complicate endodontic treatment because it can cover canal orifices and increase the risk of instrument breakage [14]. Pulp stones can obstruct the movement of files into root canals [14] and interfere with root canal irrigation and disinfection processes [15].

Studies suggested that racial and geographic differences may be the cause of the variances in frequency across various populations [9,16]. Few studies were done investigating pulp stones in Saudi subpopulations such as in Hail using periapical radiographs [17], in Abha using OPG and bitewing radiographs [18], and in Al-Jouf [3] and Madinah [19] using CBCT.

This CBCT-based study assessed the prevalence and distribution of pulp stones in the Makkah population in Saudi Arabia and evaluated their possible associations with local and systemic factors.

## Materials and methods

Study design and ethical approval

This retrospective, cross-sectional study included 390 randomly selected CBCT images from 1003 patients' electronic health records (EHR). The ethical approval was obtained from the Biomedical Research Ethics Committee of Umm Al-Qura University before starting the study (approval number: HAPO-02-K-012-2023-03-1522).

Subjects and sample size determination

The sample size was calculated by the following equation: $S=N/(1+Ne^2 )$ [20], where S=sample size, N=number of population, and e=level of precision, calculated with 5% margin error acceptance and 95% confidence level. Accordingly, the required sample was 388 participants.

The images were retrieved from the electronic health records (EHR) in the Dental Teaching Hospital at the Faculty of Dental Medicine, Umm Al-Qura University, Makkah, Saudi Arabia. They were taken for different dental purposes from January 2019 to May 2023. The inclusion criteria were as follows: CBCT images of patients at least 18 years of age and the teeth examined having fully developed roots. The exclusion criteria were as follows: CBCT images of poor quality, teeth with root canal therapy, teeth with metal crowns, resorbed roots, and unerupted teeth.

Study procedures

Selected CBCT scans were obtained using the same CBCT machine (I-CAT Vision TM, Imaging Sciences International, Hatfield, PA), set at 37.07 mAs, 120 kVp, and acquisition time=26.9 seconds. They were examined in all planes (sagittal, coronal, and axial), in both arches, for a discrete radiopaque mass in the pulp chamber or in the radicular pulp canals of all teeth, by four examiners divided into two groups. For optimal visualization, the contrast and brightness of images were adjusted using the image processing tool of the software.

The teeth were examined for tooth type and location, as well as the presence of caries, restoration, the periodontal condition, and pulp stones. Additional patient information, including age, gender, and medical condition, were obtained from their archived files. Intra- and inter-examiner stability was confirmed by training on 20 EHR before starting the study, and their consistency was measured using the kappa test, which was 91 and 89, respectively, indicating almost perfect agreement.

Statistical analysis

The collected data were statistically analyzed using a Statistical Package for Social Sciences (SPSS) software program (version 22.0) (IBM SPSS Statistics, Armonk, NY). Descriptive statistics was presented as number and percentage, a chi-square test was used for comparing the prevalence among different groups, a kappa statistics was used for the measurement of intra- and inter-examiner consistency, and a p-value of ≤0.05 was considered statistically significant.

## Results

A total of 390 patients' EHR (198 males and 192 females) were examined; the mean age of the patients was 35.63±12.33 years. The prevalence of pulp stone in individuals was 78.97%; there were statistically significant differences regarding nationality (p=0.043), age (p=0.023), and medical status (p=0.024), while the difference was nonsignificant for gender (p=0.875) (Table 1).

**Table 1:** The prevalence of pulp stone in individuals in relation to gender, nationality, age groups, and health status Similar letters (D, E, F, G, H, I, J, K, and L) or symbols (#, @, and $) indicate significant difference between corresponding groups in the same column. A chi-square test was used for comparing the prevalence CVD: cardiovascular disease

Variable		Number (%)	Pulp stone			
			Without number (%)	With number (%)	Prevalence (%)	P-value
Gender	Male	198 (50.8)	41 (10.5)	157 (40.3)	79.29	0.875
	Female	192 (49.2)	41 (10.5)	151 (38.7)	78.65	
Nationality	Saudi	266 (68.2)	63 (16.2)	203 (52.0)	76.32	0.043
	Non-Saudi	124 (31.8)	19 (4.9)	105 (26.9)	84.68	
Age groups	18-30 years	163 (41.8)	46 (11.8)	117 (30.0)	71.78^#,@,$^	0.023
	31-40 years	112 (28.7)	20 (5.1)	92 (23.6)	82.14^#^	
	41-50 years	65 (16.7)	8 (2.05)	57 (14.6)	87.69^@^	
	51 or more	50 (12.8)	8 (2.05)	42 (10.7)	84.0^$^	
Medical health	Free	299 (76.7)	49 (12.6)	250 (64.1)	84.61^D,E,F,G^	0.024
	CVD	21 (5.38)	5 (1.28)	16 (4.10)	76.19^D,H,I,J^	
	Renal disease	32 (8.21)	12 (3.08)	20 (5.13)	62.50^E,H,K^	
	Diabetes mellitus	14 (3.59)	5 (1.28)	9 (2.31)	64.29^F,I,L^	
	Others	24 (6.15)	11 (2.82)	13 (3.33)	54.17^G,J,K,L^	
Total		390 (100)	82 (21)	308 (79.0)	78.97	

Table 2 shows significant association between pulp stone prevalence and nationality (p=0.003) and age (p=008) and no association between pulp stone prevalence and health status (p=0.111) or gender (p=0.876).

**Table 2:** The association between different variables and pulp stone

Variable	Linear-by-linear association value (p-value)
Gender	0.25 (0.876)
Nationality	3.219 (0.003)
Age	7.055 (0.008)
Health status	0.122 (0.111)

The total prevalence of pulp stone in all examined teeth was 15.92% (1644 from 10326). In comparison between males and females, the prevalence was 16.02% for males and 15.82% for females with no significant difference between them (p=0.162). There were no significant differences in pulp stone prevalence, between genders, for sound (p=0.063), carious (0.054), periodontally healthy (p=0.436), and periodontally affected teeth (p=0.301), but the difference was significant for restored teeth (p=0.006) (Table 3).

**Table 3:** The prevalence of pulp stones in teeth in relation to tooth condition and gender The p-value was calculated using the chi-square test. Similar letters (A, B, C, D, E, F, G, H, and I) indicate significant difference between corresponding groups in the same column

Tooth condition	Male, number (%)	Female, number (%)	Total, number (%)	P-value
Teeth without pulp stones (PS)	4268 (41.33)	4414 (42.74)	8682 (84.08)	0.162
Teeth with PS	814 (7.88)	830 (8.04)	1644 (15.92)	
Total	5082 (49.22)	5244 (50.78)	10326 (100.00)	
Prevalence of PS	16.02	15.82	15.92	
Pulp stones in relation to dental caries and restorations				
Sound teeth	2095 (41.52)	2480 (49.15)	4575 (90.67)	0.063
Sound teeth with PS	242 (4.79)	229 (4.54)	471 (9.33)	
Total	2337 (46.32)	2709 (53.69)	5046 (100.00)	
Prevalence	10.36^A,B^	8.45^D,E^	9.33^G,H^	
Decayed teeth	1898 (42.50)	1654 (37.04)	3552 (79.53)	0.054
Decayed teeth with PS	474 (10.62)	440 (9.85)	914 (20.47)	
Total	2372 (53.11)	2094 (46.89)	4466 (100.00)	
Prevalence	19.98^A,C^	21.01^D,F^	20.47^G,I^	
Restored teeth	275 (33.78)	280 (34.40)	555 (68.18)	0.006
Restored teeth with PS	98 (12.04)	161 (19.78)	259 (31.82)	
Total	373 (45.82)	441 (54.18)	814 (100.00)	
Prevalence	26.27^B,C^	36.51^E,F^	31.82^H,I^	
P-value	0.000	0.000	0.000	
Pulp stones in relation to periodontal condition				
Periodontally healthy	3677 (41.21)	3869 (43.36)	7546 (84.58)	0.436
Periodontally healthy with PS	677 (7.59)	699 (7.84)	1376 (15.42)	
Total	4354 (48.80)	4568 (51.20)	8922 (100.00)	
Prevalence	15.55	15.30	15.42	
Periodontally affected	591 (42.09)	545 (38.82)	1136 (80.91)	0.301
Periodontally affected with PS	137 (9.76)	131 (9.33)	268 (19.09)	
Total	728 (51.85)	676 (48.15)	1404 (100.00)	
Prevalence	18.82	19.38	19.09	
P-value	0.047	0.023	0.031	

According to tooth condition, there were significant differences between pulp stone prevalence of sound and carious and restored teeth and between carious and restored teeth for both males (p=0.000) and females (p=0.000), as well as total prevalence (p=0.000). Regarding periodontal condition, there was a significant difference between pulp stone prevalence of periodontally affected teeth and periodontally healthy teeth for males (p=0.047) and females (p=0.023) and total prevalence (p=0.031) (Table 3).

**Figure 1:**
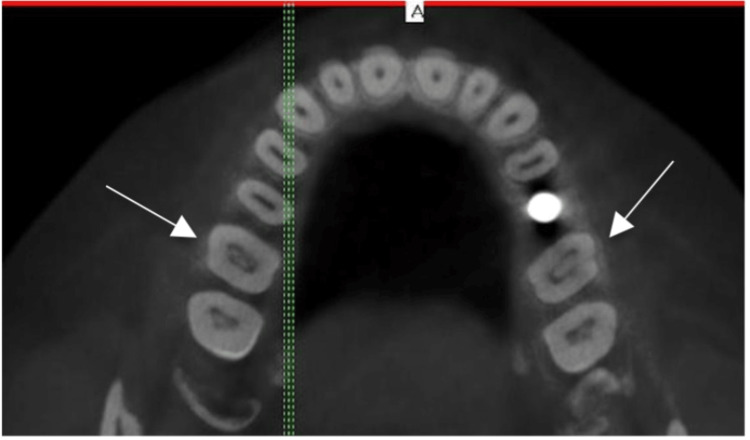
CBCT axial section showing pulp stones in the upper right and left first molars CBCT: cone-beam computed tomography

**Figure 2:**
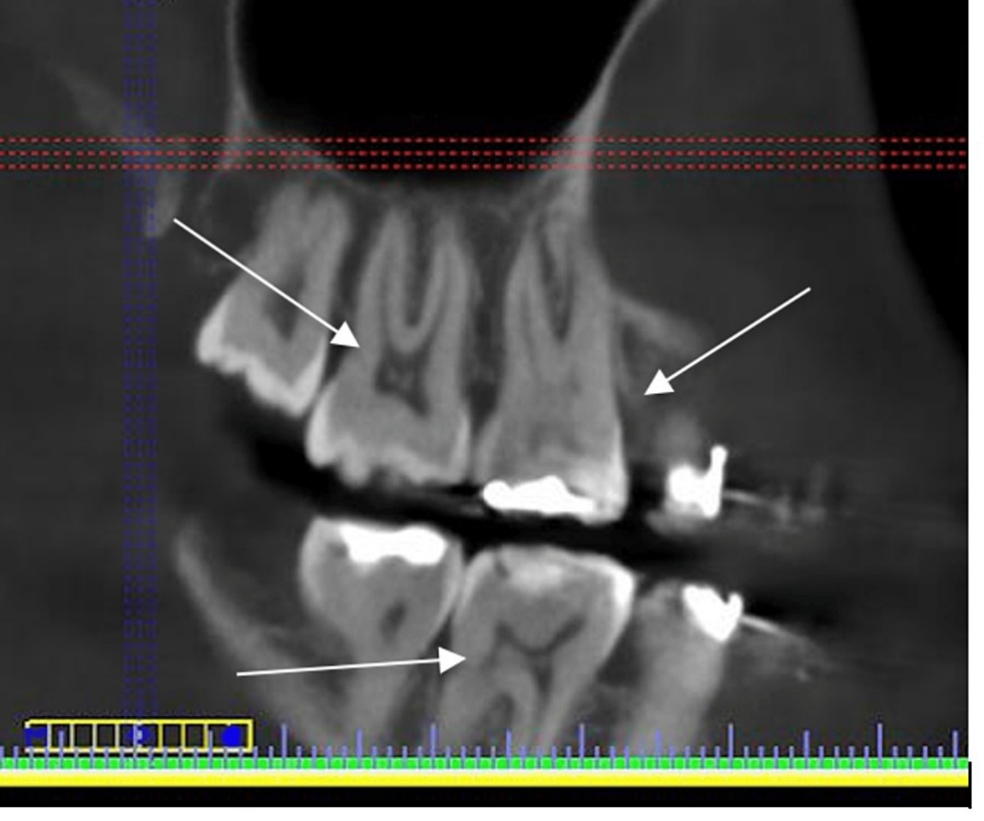
CBCT sagittal section showing pulp stones in the upper first and second molars and lower first molar CBCT: cone-beam computed tomography

**Figure 3:**
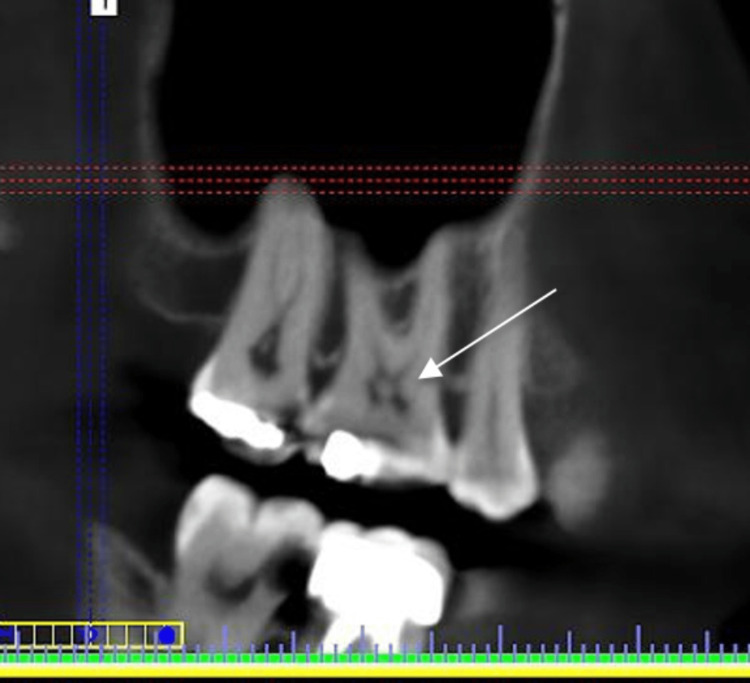
CBCT sagittal section showing pulp stone in the upper first molar CBCT: cone-beam computed tomography

The highest total pulp stone prevalence was found in the first molar (46.59%), followed by the second molar (34.18), third molar (19.79), second premolar (7.56%), first premolar (6.56%), canine (4.75%), lateral incisor (2.12%), and central incisor (1.54%) (Figures 1-3). In relation to tooth condition, the first molar had the highest prevalence of pulp stone in sound (67.81%), decayed (47.87%), and restored teeth (43.15%), while the lateral incisor had the lowest prevalence. Regarding the pulp stone prevalence among periodontally affected teeth, the first molar tooth had the highest prevalence (46.61%), followed by the second molar (35.05%), third molar (18.20%), second premolar (7.93%), first premolar (6.82%), canine (4.58%), lateral incisor (1.65%), and central incisor (1.52%) (Table 4).

**Table 4:** The prevalence of pulp stone in relation to different tooth conditions for each tooth type Similar letters (E, K, O, R, and S) indicate significant difference between corresponding groups in the same column UR, upper right; UL, upper left; LR, lower right; LL, lower left

Tooth		Dental caries and restorations			Periodontal condition		Total prevalence, %
		Sound, %	Carious, %	Restored, %	Affected, %	Healthy, %	
Third molar	UR	15.54	19.32	60.00	12.81	15.38	17.37
	UL	13.64	20.83	25.00	17.26	35.29	18.92
	LR	15.91	29.87	45.45	18.44	44.44	25.77
	LL	17.44	16.47	50.00	23.86	48.00	22.06
Total		15.49	18.06	40.00	18.20	38.50	19.79
Second molar	UR	32.71	38.50	41.76	37.03	34.55	36.68
	UL	44.20	13.59	44.87	34.48	33.87	33.14
	LR	29.03	34.66	41.38	34.34	37.50	34.78
	LL	31.96	34.24	39.22	34.25	27.69	33.25
Total		35.40	32.43	42.15	35.05	28.77	34.18
First molar	UR	64.18	53.40	47.69	54.44	38.37	51.18
	UL	67.92	50.24	47.06	52.42	38.79	48.43
	LR	72.31	42.86	42.11	43.93	24.29	44.54
	LL	58.33	43.21	35.70	35.66	20.55	39.09
Total		67.81	47.87	43.15	46.61	31.88	46.59
Second premolar	UR	5.17	6.86	26.09	7.64	2.22	6.28
	UL	4.20	4.82	13.04	5.19	3.92	5.01
	LR	6.45	7.74	25.00^O^	11.25	3.57	7.24
	LL	9.09	13.02	12.50^O^	7.55	9.68	11.11
Total		6.38	8.11	19.35	7.93	4.52	7.56
First premolar	UR	5.17	9.50	13.33^S^	8.07	7.25	7.92
	UL	1.55	6.10	0.00^S^	3.77	2.38	3.48
	LR	4.78	8.39	28.57	7.97	11.11	6.90
	LL	4.76	11.64	25.00	6.69	11.76	8.14
Total		4.22	8.86	10.91	6.82	5.85	6.56
Canine	UR	3.61	6.89	11.76^R^	4.49	13.79^K^	5.20
	UL	3.18	8.69	24.14^R^	5.59	5.00^K^	5.54
	LR	3.46	15.38^E^	0.00	4.01	8.33	4.38
	LL	3.76	7.41^E^	0.00	4.26	0	3.90
Total		3.51	8.70	18.00	4.58	8.33	4.75
Lateral incisor	UR	1.64	8.69	0.00	1.98	0.00	1.89
	UL	0.66	11.54	0.00	1.40	10.00	1.64
	LR	1.36	0.00	0.00	1.87	0.00	1.33
	LL	1.52	0	0.00	1.35	0	1.58
Total		1.34	7.14	0.00	1.65	2.56	2.12
Central incisor	UR	1.65	8.33	9.38	2.59	0.00	2.44
	UL	1.68	11.76	2.50	2.25	4.17	2.38
	LR	1.90	0.00	0.00	1.35	0.00	0.63
	LL	1.89	0.00	0.00	1.89	0.00	1.83
Total		1.79	9.09	5.26	1.52	1.69	1.54

In comparison between maxillary and mandibular teeth, there was no significant difference between them for total pulp stone prevalence (p=0.392). In comparison between the right and left sides, there was no significant difference between them for total pulp stone prevalence (p=0.222) (Table 5).

**Table 5:** Comparison between the prevalence of pulp stone in the right- and left-side maxillary and mandibular teeth The p-value was calculated using the chi-square test

Variables		Total prevalence, %
Position	Maxillary	14.90
	Mandibular	14.43
	P-value	0.392
Side	Right	16.59
	Left	15.31
	P-value	0.222

## Discussion

Pulp stones are discrete calcifications that may exist freely within the pulp tissue or be attached to or embedded in dentine. Their presence complicates the nonsurgical endodontic treatment by hindering access to root canals and their subsequent shaping [4]. In the current study, we used CBCT for the identification of pulp stones. This technique overcomes the limitations of conventional radiographic modalities as it provides high-resolution, three-dimensional images of the teeth and related anatomical structures, with better specificity and accuracy. It has many beneficial uses in endodontics as it allows to viewing of anatomical details in coronal, axial, and sagittal planes without the superimposition of structures [21]. It is an efficient noninvasive technique in locating and assessing pulp stones in clinical investigations [5,22,23].

Pulp stone prevalence has a vast range in the literature, from 8% to 90% [24]. The total prevalence of pulp stones in the current study was 78.97% of the subjects and 15.92% of the examined teeth. It was higher than the prevalence reported in other Saudi subpopulations. In Riyadh, Al-Nazhan and Al-Shamrani [25] detected pulp stones in 10.2% of the inspected teeth. In the Al-Jouf region, Patil et al. [3] stated that 50.93% of patients and 13.34% of teeth had pulp stones. In Hail, Sadoon et al. [17] reported the prevalence in 28% of the individuals and 12% of the teeth; in Abha, Alaajam et al. [18] described pulp stones in 14.7% of the patients and 4.6% of the teeth, while in Madinah, the prevalence was 26% of the individuals and 9.2% of the teeth [19]. A systematic review assessing the prevalence of pulp stone in Saudi Arabia found that it ranged from 4.6% to 50.93% among the population and between 10.2% and 13.34% in the assessed teeth [24]. However, Kaabi et al. [23], in Riyadh, reported a higher prevalence, 98.3% of the examined dental arches and 52.1% of the teeth.

Our findings were also higher when compared to studies from different areas of the world. Hamasha and Darwazeh [26] detected pulp stones in 51% of the patients and 22% of the teeth in Jordanians; in Australians, Ranjitkar et al. [9] reported the prevalence in 46% of the individuals and 10% of the teeth. In the Northern Indian Central Punjabi population, Bains et al. [12] observed pulp stones in 41.8% of the subjects and 9.09% of the teeth. In Malaysia, Kannan et al. [27] identified pulp stones in 44.9% of the subjects and 15.7% of the teeth. In Brazil, the prevalence was 31.9% of the patients and 9.5% of the teeth [22]. In Yemen, Kalaji et al. [28] described pulp stones in 18.6% of the individuals and 3.99% of the teeth. In Turkey, Tassoker et al. [5] identified pulp stones in 52% of the subjects and 7.7% of the teeth, while Sezgin et al. [29] found that 24.2% of the patients and 3.3% of teeth had at least one pulp stone. In Libya, Alawjali [30] discovered pulp stones in 30.2% of the patients and 8.4% of the teeth. However, a study by Hsieh et al. [13] on a Taiwanese population revealed a higher prevalence of pulp stones, than the present study, in 83.3% of the subjects and 31.3% of the of the teeth.

In the current study, pulp stones were assessed in both the pulp chamber and root canals, which may contribute to the high prevalence reported, while many studies evaluated only the coronal pulp [9,12,18,25,26,29,30]. Another factor that may affect the findings is the method of examination used, whether CBCT, conventional radiograph, or histology [24]. Several studies used bitewing radiographs [9,12,18,25,26], periapical radiographs [17,26,27], and OPG [18,28,30]. The two-dimensional radiographic techniques have limitations that might underestimate the actual incidence of pulp stones as they only detect calcified structures larger than 200 μm in diameter, meaning that the true prevalence of pulp stones is probably higher [27,31]. It should be emphasized that although CBCT can provide a true pulp stone prevalence, its routine use for the detection of pulp stones should not be encouraged due to higher radiation dose compared to two-dimensional radiography [21,22]. According to the American Association of Endodontists (AAE) and the American Academy of Oral and Maxillofacial Radiology (AAOMR) joint position statement, CBCT should not be routinely used for screening needs or for endodontic diagnosis if there are no clinical signs and symptoms. Its use should be reserved only when the imaging purpose cannot be achieved by a lower-dose two-dimensional radiograph [32]. And when used, it is mandatory that the radiation exposure is held as low as reasonably practicable (ALARP) [21]. Although histological examination is more accurate and can elucidate a higher number of pulp stones, it is an invasive technique that restricts its use; in addition, the limited number of histological sections through each tooth may result in underreporting [4,24]. The sample size and the type of teeth evaluated are important factors; some authors included only molars and premolars in their researches [9,18,25,28,30], while the current study assessed all teeth types. Ethnic and geographic variations also affect pulp stone's occurrence [11,16,26,30].

In the present study, a significant association was found between pulp stone prevalence and nationality; this could be attributed to the effect of racial variations and geographic differences as suggested by Çolak et al. [16]. Also, a positive correlation between pulp stone prevalence and age was revealed; this was consistent with previous studies [5,16,17,19,33]. Other investigators reported no relation between age and pulp stone frequency [10,23,27,28,34]. Regarding the health condition, the prevalence, in the current study, was significantly higher in healthy subjects, suggesting no correlation between patient health condition and pulp calcification. This differs from the literature where medically compromised patients were reported to develop more likely pulp stones [6,33,35]. The small number of patients with medical conditions included in the present study might be a possible reason affecting the comparisons between patients with medical problems and healthy subjects. Among the medical conditions encountered in this study, the prevalence of pulp stones was significantly higher in patients with cardiovascular diseases (CVD) as reported by other studies [35,36]. A systematic review and meta-analysis found a significant association between pulp stone and CVD [37]. A possible explanation was proposed by Edds et al. who suggested that the pathogenesis of dental pulp calcification and calcified atheromas might be similar and that the etiology of the calcification of large and small vessels is the same [6].

There was no significant association between gender and pulp stone incidence. This finding agreed with several studies [5,9-11,13,17,19,22,26-28,38-42] and disagreed with others that reported a higher incidence in males [3,23,26] or found a higher prevalence in females [12,16,18,19,25,29,30,34].

According to teeth condition, the present study showed a significant correlation between the prevalence of pulp stones and restored (31.82%) and carious teeth (20.47%), with a more significant occurrence in restored teeth. Different studies emphasize the role of chronic irritating factors in pulp calcification [3,5,12,17,23,24,27]. Researchers reported that pulp stones prevailed in restored and carious teeth more than sound teeth [5,19,22,27,38] with even higher incidence when both caries and restoration exist [23,27]. Tassoker et al. [5] detected a direct relation between the probability of pulp stone existence and the increased depth of restorations; they explained that chronic irritation of pulp tissue led to pulp stone production as a defense reaction. However, Sezgin et al. [29] found that pulp stones were more commonly found in teeth with medium-depth restorations. In contrast to the previous findings, Gulsahi et al. [42] found no significant association between caries or restorations and the occurrence of pulp stones. Similarly, Alawjali [30] detected more pulp stones in the intact teeth compared to carious and restored teeth.

Our findings indicated a significantly higher prevalence of pulp stones among periodontally affected teeth; these findings consisted of previous studies showing a close correlation between pulp calcifications and periodontal disease [11,12,43,44]. Sheykhrezaee et al. [43] stated that periodontal disease can produce fibrosis and calcification of the dental pulp. Yemenoglu et al. [44] concluded that pulp stones were associated with advanced periodontal pathosis. Bains et al. [12] found that 16.41% of the teeth with pulp stones had periodontal involvement. Conversely, Mirah et al. [19] reported that the relation between pulp stones and periodontal pathology was seldom significant.

Regarding the type of teeth, in the current study, pulp stones prevailed in the molars as reported previously in the literature [3,17-19,22]. The prevalence was highest in the first molars (46.59%), followed by the second molars (34.18%), third molars (19.79%), first and second premolars, and then canines, and the lowest prevalence was found in the central incisors (1.54%). A similar pattern was reported by Sezgin et al. [29] in Turkey; pulp stones were more common in the molars and then the premolars and then the canine and anteriors. In Madinah, Saudi Arabia, Mirah et al. [19] reported the highest prevalence of pulp in the molars, followed by the anteriors and least in the premolars; similar pattern was described by Kaabi et al. [23] in another Saudi population. Chalikkandy et al. [24], in their systematic review of pulp stone prevalence in Saudi Arabia, found it to be higher in the molar teeth compared to the premolars. A potential explanation for the increased frequency of pulp stones in the molars relates to their larger size and better supply of blood to the pulp tissues, predisposing to more precipitation of calcifications [26]. Similar to our findings, many authors reported a higher prevalence of pulp stones in the first molars than in the second molars [9,12,13,17,25,26,30,45]. This was explained by the early eruption of the first molar, so it is exposed to more degenerative changes, confirming that pulp calcification increases with age [25].

In comparison between maxillary and mandibular teeth, there was no significant difference between them for total pulp stone prevalence. This was in accordance with several studies held in various populations [3,5,22,26,27,42,46]. Our findings disagree with those of Alaajam et al. [18] and Kaabi et al. [23] in Saudi Arabia, Kalaji et al. [28] in Yemen, Alawjali [30] in a Libyan population, Hsieh et al. [13] in a Taiwanese population, Sezgin et al. [29] and Çolak et al. [16] in a Turkish population, and Bains et al. [12] in an Indian population; they found that pulp stone prevalence was higher in the maxillary than mandibular teeth. In contrast, Ravichandran and Vadivel [10] found that pulp stones were more frequent in the mandible in India. In the current study, there was no significant difference between the right and left sides for total pulp stone prevalence. Our findings agreed with many researchers [3,13,16,22,30] and disagreed with others [18,19,28].

## Conclusions

The prevalence of pulp stones in the Makkah population is high. A positive association was found between nationality, age, tooth restorations, caries, periodontal diseases, and pulp stone prevalence, but no correlation was found with patients' health or gender. The molars were the most affected teeth, while the incisors were the least. No significant difference existed in the frequency of pulp stones between the maxillary and mandibular teeth nor between the right and left sides.

Dental practitioners can make the necessary adjustments to the standard root canal treatment protocol for handling such teeth by using the observations from this study.
